# Geospatial Analysis of Persons with Movement Disorders Living in Underserved Regions

**DOI:** 10.5334/tohm.635

**Published:** 2021-08-20

**Authors:** Alaina Giacobbe, Ka Loong Kelvin Au, Oliver T. Nguyen, Kathryn Moore, Emily Dinh, Adolfo Ramirez-Zamora, Michael S. Okun, Leonardo Almeida

**Affiliations:** 1 University of Pittsburgh Medical Center, Department of Neurology, Pittsburgh, Pennsylvania, US; 2 University of Florida, Department of Neurology, Gainesville, Florida, US

**Keywords:** geography, spatial analysis, Movement Disorders, underserved

## Abstract

**Background:**

Movement disorders persons from underserved areas have increased barriers to access tertiary care. There is currently limited data on the geographic and demographic profile of movement disorders persons from underserved areas.

**Methods:**

A retrospective chart review of the geographic and demographic profile of consecutive cases seen between 2002–2017 at the University of Florida Norman Fixel Institute for Neurological Diseases (UF-NFIND) was performed. Information collected included age, sex, diagnosis, zip code, treatment received, and insurance information. The distances between each person’s home residence and the nearest movement disorders center of excellence (MDC) as well as the distance to the UF-NFIND were calculated using ArcGIS 10.3.

**Results:**

A total of 5.2% (355/6867) of the sample population were identified as a Medicaid/self-pay population and classified as underserved. The most common diagnoses were tic disorder (19.2%), dystonia (18.3%), and Parkinson’s disease (14.3%). In underserved persons, the median distances from their homes to the UF-NFIND (82.19 [45.79–176.93] km) vs. their nearest MDC (63.34 [26.91–121.43] km) were significantly different (*p* < 0.001).

**Discussion:**

Underserved persons in our study travelled further to receive subspecialty care at UF-NFIND than closer MDCs. Potential reasons for underutilization of closer care could possibly include research opportunities, availability of specific treatments or procedures, insurance restrictions, and limited specialist availability. Despite this observation, underserved persons were underrepresented at our institution compared to the proportion of Medicaid/uninsured patients in Florida. Our results highlight the need for increased awareness of care options for underserved movement disorders populations.

## Introduction

Movement disorders include a complex group of neurological conditions characterized by excessive (hyperkinetic) or reduced (hypokinetic) movements [1]. These disorders may present with a variety of motor symptoms including tremors, parkinsonism, dystonia, chorea, myoclonus, and tics. Individuals may also experience non-motor comorbid symptoms such as cognitive, psychiatric, and autonomic dysfunction. The presence of these features typically add to disability and can impair quality of life [1]. Movement disorders often require a higher level of clinic-based care beyond a simple specialty consultation (i.e., neurologist). The needs of a person with movement disorders commonly includes utilization of multidisciplinary care with physical therapy (PT), occupation therapy (OT), speech language pathology (SLP), nursing, neuropsychology, psychiatry or social work services.

Movement disorders centers of excellence (MDC) are designed to provide specialized multidisciplinary management which can be beneficial for Parkinson’s disease cases and for several other movement disorders [2,3,4]. MDCs provide access to advanced treatment options, including botulinum toxin injections, continuous medication infusions or pump therapy, implants such as deep brain stimulation (DBS) and access to experimental treatments through clinical trials [5,6].

Access to specialized tertiary centers remains a critical problem in global healthcare systems. In the United States, many movement disorders persons lack access to a MDC and many are underserved, which we defined as insurers of Medicaid or self-pay. Medicaid is a joint federal and state government funded insurance program to provide health insurance to low-income adults, children, pregnant women, elderly adults and people with disabilities who otherwise would not be able to afford private health insurance. Self-pay patients are usually low-income, vulnerable patients with no insurance who rely on “safety-net” institutions, like the University of Florida, that provide healthcare for individuals regardless of insurance status.

Most studies have failed to quantify access disparity. In the current study we sought to identify clinical and demographic characteristics of underserved persons with movement disorders. Establishing geospatial correlations to care will guide potential improvement in the field.

## Methods

### Study Design and Patient Population

This was a retrospective study using data collected at an academic tertiary MDC for visits between January 2002 and June 2017. The study was approved by University of Florida’s institutional review board. This institution is home of the INFORM clinical database, which includes longitudinal information of nearly 13,000 persons who consented to having their clinical information stored for research purposes, including retrospective data analysis or screening for clinical trials. An initial query of clinic encounters was conducted to identify persons seen at our location. Their medical record numbers, payor and zip code of primary residence were obtained. For underserved individuals (i.e., insurer of Medicaid or self-pay), additional information was cross-referenced within the INFORM database, including diagnosis, demographics, disease-specific data, and types of treatment received. The type of treatment was defined as clinical visit (with neurology), multidisciplinary rehabilitation (PT, OT, SLP), botulinum toxin injection, or DBS programming visit.

### Definition and Identification of Movement Disorders Centers of Excellence

MDCs were arbitrarily defined based on: 1) availability of two or more movement disorders-trained neurologists at a given location, 2) presence of multidisciplinary rehabilitation, and 3) presence of research opportunities and clinical trials for movement disorders persons.

An initial search under the Movement Disorders Specialist Directory at the International Parkinson’s and Movement Disorders Society online portal was conducted [7]. Each provider listed for the state of Florida had his/her addresses of practice and affiliations confirmed by a subsequent online search. Identified group practices that met all three criteria above were classified as MDCs.

### Spatial and Statistical Analyses

**Figure 1:**
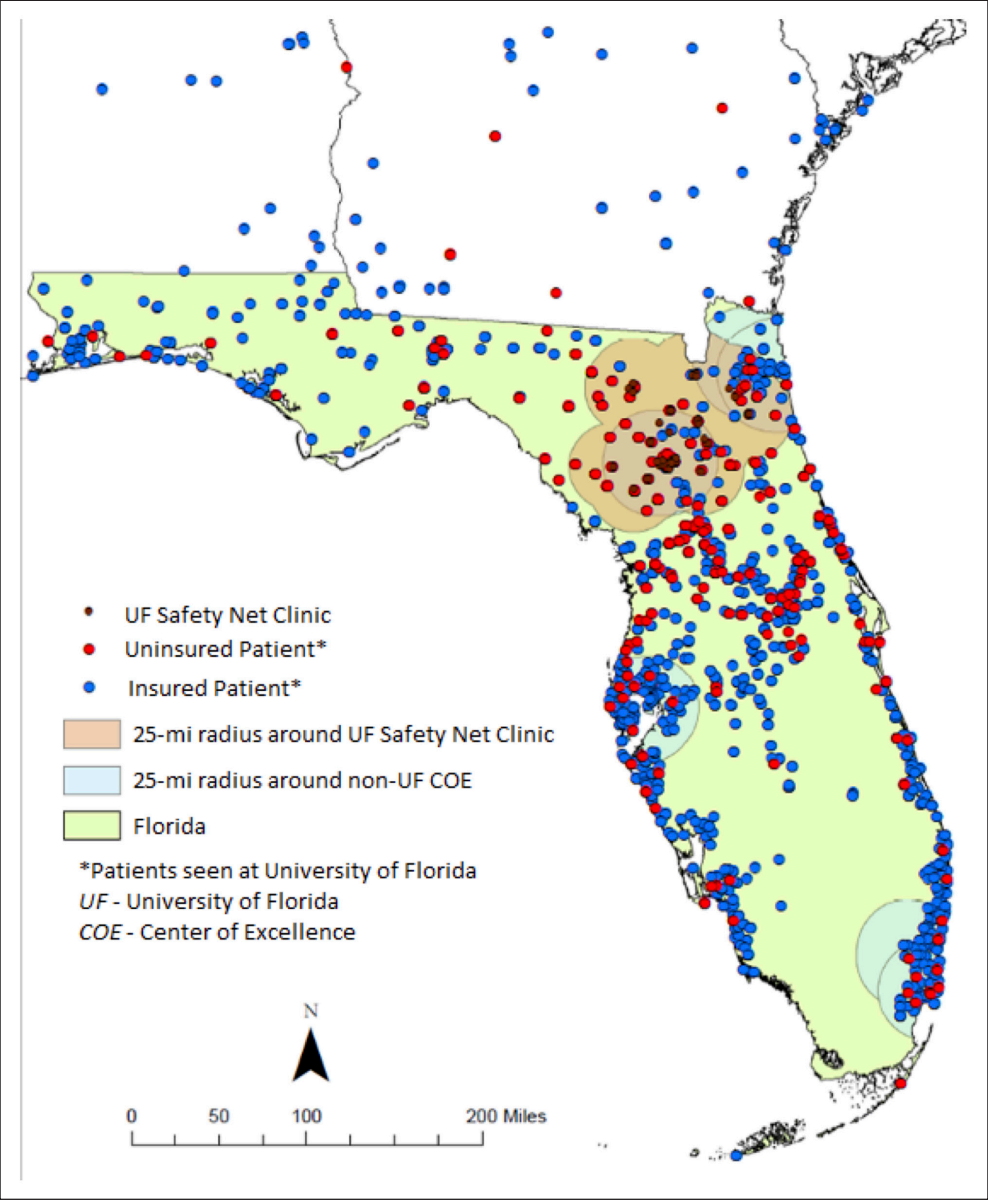
**Spatial Analysis of patients seen at the UF-NFIND.** Geographical distribution of insured and uninsured persons seen at the UF-NFIND and their relationship to the nearest movement disorders center of excellence.

Spatial trend analyses were conducted using ArcGIS 10.3 software. People located in Florida and the surrounding states of Alabama, Georgia, and South Carolina were included. Dot distribution maps were created by plotting the home zip codes of persons traveling to the UF-NFIND, including insured and uninsured persons, with one dot corresponding to one patient (***Figure 1***). The catchment areas shown for MDCs located in Florida and the UF safety net clinics (geared towards underserved persons) were defined as a 25-mile radius.

Subsequent quantitative statistical analyses conducted for underserved persons showed categorical variables (diagnoses and treatment types) as counts and percentages. Disorders or conditions containing fewer than five persons were excluded from the subgroup analysis to limit the type II small sample size error.

The straight-line distances each person traveled from home to their nearest MDC and to UF were presented as medians with interquartile ranges. For the distance comparisons, the Shapiro-Wilk test of normality resulted in p-values <0.05. Therefore, unpaired Wilcoxon rank sum (Mann-Whitney U) tests were implemented to compare distances from insured vs. uninsured patient’s residencies to UF-NFIND and the nearest MDC. Next, paired-samples Wilcoxon signed-rank tests were conducted to compare differences in median distances to UF-NFIND and their nearest MDC for uninsured patients by diagnosis and treatment received. α = 0.05 was assumed for a level of statistical significance for all statistical tests. R statistical software (R Core Team, Vienna, Austria) was used to perform all statistical analyses.

## Results

### Population Characteristics

A total of 6,867 persons with complete datasets were identified across all payors (e.g., Medicare, commercial insurance, Medicaid, and self-pay). Of these, 355 (5.2%) persons were classified as underserved. Geospatial representation of insured and uninsured persons seen at UF-NFIND is shown in ***Figure 1***.

Among the underserved persons seen at the UF-NFIND, half of these persons were male, and the mean age was 40.0 ± 20.4 years. The most common diagnoses were tic disorders (19.2%), followed by dystonia (18.3%) and Parkinson’s disease (PD) (14.3%). Greater than a third (34.5%) of these persons received at least one rehabilitation service (PT, OT and/or SLP), 11% received botulinum toxin injections, and 6% were followed for management of DBS. Clinical and demographic characteristics of the underserved population is summarized in ***Table 1***.

**Table 1:** Patient Demographics of Underserved Movement Disorder Persons.


DIAGNOSIS	N	MEDICAID	UNINSURED	FEMALE (%)	MEAN AGE (YEARS) +/– SD	REHABILITATION (%)	BOTULINUM TOXIN (%)	DBS (%)

Tic disorders	67	64	3	22 (32.8)	19.9 +/– 11.6	28 (41.8)	0 (0.0)	1 (1.5)

Dystonia	65	46	19	41 (63.1)	42.6 +/– 18.2	17 (26.2)	34 (52.3)	10 (15.4)

Parkinson’s disease	52	20	32	23 (44.2)	65.2 +/– 11.1	29 (55.8)	3 (5.8)	10 (19.2)

Essential Tremor	41	33	8	18 (43.9)	53.1 +/– 13.6	11 (26.8)	1 (2.4)	1 (2.4)

Tremor	35	30	5	24 (68.6)	45.3 +/– 17.6	5 (14.3)	0 (0.0)	1 (2.9)

Ataxia	25	16	9	15 (60.0)	46.0 +/– 16.4	10 (40.0)	1 (4.0)	0 (0.0)

Huntington’s disease	17	12	5	7 (41.2)	45.9 +/– 13.0	12 (70.6)	0 (0.0)	0 (0.0)

Parkinsonism NOS	10	7	3	2 (20.0)	62.4 +/– 12.1	2 (20.0)	0 (0.0)	0 (0.0)

Myoclonus	8	7	1	5 (62.5)	43.1 +/– 13.6	0 (0.0)	0 (0.0)	0 (0.0)

Stereotypies	7	7	0	1 (10.0)	31.7 +/– 20.3	0 (0.0)	0 (0.0)	0 (0.0)

Tardive syndromes	7	5	2	5 (71.4)	48.3 +/– 13.1	1 (14.3)	2 (28.6)	0 (0.0)

Chorea	5	5	0	4 (80.0)	44.8 +/– 14.4	1 (20.0)	0 (0.0)	0 (0.0)

Other	16	9	7	9 (56.3)	52.25 +/– 21.5	6 (37.5)	0 (0.0)	0 (0.0)

All patients	355	261	94	176 (49.6)	44.4 +/– 20.5	122 (34.4)	41 (11.5)	23 (6.5)


SD – Standard deviation; NOS – not otherwise specified; DBS – Deep Brain Stimulation.

### Utilization of Services and Distance to Tertiary Center

Both insured and underserved persons travelled to the UF-NFIND from distances greater than their nearest MDCs to receive specialty care. For insured persons across all diagnoses, the median distance to the nearest MDC was 90.30 [29.33–159.28] km and the distance to UF-NFIND was 127.24 [61.34–247.62] km (*p* < 0.001). The median distance to the nearest MDC for underserved persons was 63.34 [26.91–141.43] km and the distance to UF-NFIND was 82.19 [45.79–176.93] km, respectively (Wilcoxon rank sum *p* < 0.001).

Wilcoxon rank sum tests by diagnosis revealed that underserved persons with Tardive syndromes travelled the furthest (226.28 [75.59–377.62] km) to reach the UF-NFIND, followed by persons with Parkinson’s disease (168.142 [48.83–234.79] km). Group comparisons showed that underserved persons with ataxia, dystonia, HD, PD, tic disorders, and tremors other than essential tremor (ET) traveled significantly longer distances to seek care at the UF-NFIND as compared to the distance of the nearest MDC to their zip code of residence (*p* < 0.05 for all conditions). ***Table 2*** summarizes distance travelled for each specific diagnosis.

**Table 2:** Average distances from Medicaid/self-pay patients to movement disorders centers according to disease type.


DIAGNOSIS	N (%)	DISTANCE TO NEAREST CENTER, KM MEDIAN (IQR)	Distance to UF, Km Median (IQR)	P-VALUE^†^

Ataxia	25	99.76 (54.69–219.18)	165.85 (69.55–328.48)	0.003*

Chorea	5	77.033 (38.433–221.440)	80.004 (38.43–221.44)	1

Dystonia	65	70.70 (27.79–158.95)	119.76 (61.53–218.23)	<0.001*

ET	41	62.53 (38.43–81.17)	62.53 (38.43–91.11)	0.096

HD	17	71.61 (52.39–144.17)	159.01 (71.61–182.36)	0.014*

Myoclonus	8	51.553 (34.59–74.00)	59.649 (44.61–99.74)	0.371

PD	52	70.580 (25.68–148.98)	168.142 (48.83–234.79)	<0.001*

Park. NOS	10	58.611 (9.25–76.47)	66.06 (18.19–118.23)	0.371

Stereotypies	7	6.016 (4.26–23.12)	6.016 (4.26–104.80)	0.371

Tardive	7	81.90 (50.52–362.6)	226.28 (75.59–377.62)	0.371

Tic disorders	67	63.31 (30.12–100.78)	69.66 (44.60–119.72)	0.003*

Tremor	35	42.724 (6.562–74.52)	61.138 (10.28–87.90)	0.009*


ET: Essential Tremor. HD: Huntington Disease. PD: Parkinson’s Disease. NOS: Not-otherwise specified. ^†^ Paired-samples Wilcoxon (signed-rank) p-value. * Comparisons found to be statistically significant.

Underserved persons receiving DBS travelled the furthest, with a median of 181.63 [90.13–534.46] km from their zip codes to UF. Subgroup comparisons revealed longer travel distances from home zip code to UF-NFIND in comparison to their nearest MDC for rehabilitation services (66.33 [26.76–118.98] km vs. 84.99 [43.85–172.15] km, *p* < 0.001), specialized treatment with botulinum toxin injections (71.61 [31.05–121.43] km vs. 100.51 [45.79–164.07] km, <0.001) and DBS management (140.89 [27.97–168.60] km vs. 181.63 [90.13–534.46] km, *p* < 0.001).

## Discussion

To our knowledge, this is the first study assessing the access to specialized tertiary care for underserved individuals suffering from movement disorders. One of the most striking findings was that only 5.2% of all persons regardless of diagnosis who were followed at the UF-NFIND were identified as underserved. This percentage of underserved persons was much lower when contrasted with an estimated 19.5% of the Florida general population enrolled on Medicaid and the 14.9% of uninsured persons that was reported in 2017 [8,9]. These data raise concerns for potential barriers to accessing specialized neurology care and provides an opportunity to identify plausible factors accounting for these observations. Our findings are relevant since the UF center has been classified as a safety net institution (i.e. dedicated mission to deliver care for underserved persons residing within a large surrounding catchment area). Therefore, a much greater proportion of underserved persons would be expected to be evaluated in our clinic if access was adequate. These results are in agreement with the findings from Timbie et al. (2019) who reported that neurology was one of four specialties where access for Medicaid were labelled as challenging [10].

A variety of factors might explain the smaller proportion of underserved persons in our clinic cohort, including access to a referral, administrative and financial barriers, caregiver burden, travel time and travel cost. Underserved persons in Florida are required to be enrolled with a primary care physician (PCP) and it is the PCP who initiates a referral to subspecialty care. Many persons unfortunately do not have easy access to a PCP. Based on the 2014 National Health Interview Survey (NHIS), Florida was one of nine states with a higher percentage of adults without a usual place for accessing primary medical care (21.5%) as compared to the national average (17.3%) [11]. Further analysis of the data revealed that the main barriers to specialty care access for Medicaid persons included: 1) specialists/practices accepting new persons (69.4%) and/or 2) administrative requirements affecting access to specialist consults (49.0%). Lower income families and uninsured had a lower likelihood of seeing a neurologist [12]. Lower socioeconomic status has been tied to higher rates of transportation barriers [13], and this factor likely affects persons seen at MDCs who frequently have chronic conditions.

Further, our results demonstrate that underserved persons will travel greater distances to receive specialized care and procedures at tertiary centers (UF-NFIND) despite closer proximity to other facilities. Potential explanations include unavailability of specific services at their local MDC, seeking second opinions from sub-specialists within the field of movement disorders (e.g. specialized expertise in tics, or dystonia) or institution-specific financial assistance programs. Another reason could be the larger number of clinical trials available at tertiary centers, like UF-NFIND, which allows patients to get access to medications and transportation at no or reduced cost. Additionally, our results cannot account for persons who receive co-managed care between different institutions (i.e. receiving care from a local movement disorders specialist/MDC and periodically being seen at another MDC). Finally, it is possible that a comparable scenario to our results is encountered at the other MDCs in our state, where persons closer to the UF-NFIND will travel greater distances to be seen at a different MDC. Those results cannot be supported by the data available to us and thus would warrant future multicentered studies. Nonetheless, our results may reflect the overall high demand of specialized care in a movement disorders population, in which the high patient-to-provider ratio will result in increasing wait times, leading persons to travel to see the first available provider. It is conceivable that greater access discrepancies could be uncovered within the underserved population. Further studies could focus on assessing needs and addressing potential solutions to accessing specialized care.

Although ET and PD represents the two most common movement disorders in an adult population [1,14], dystonia and tic disorders were the most commonly represented in the data from our underserved population. This inconsistency may represent the majority of Florida Medicaid enrollment (50%) being in the 0–18 age group [11]. Both tic disorders and several genetic and acquired forms of dystonia have onset of symptoms before the age of 18 [15,16]. In comparison, the typical age of onset of PD is in the sixth decade of life [15,17].

## Study Limitations

Our study concentrated on persons evaluated at one tertiary MDC who consented to share their information in our clinical database, limiting randomization, which makes our study vulnerable to selection bias and limits the generalizability of our results. Additionally, our choice to use insurance status to define “underserved” patients focuses on socioeconomic status, and may not include other vulnerable populations, like racial and ethnic minorities, LGBTQ+, the chronically ill and disabled, and those with poor education. Future studies are needed to explore how these other factors affect access to movement disorder’s neurologists.

Our definition of MDC included two or more movement disorders physicians located in one practice, and thus this definition did not account for practices with a single movement disorders specialist using an allied healthcare team. Furthermore, the decision to use a 25-mile-radius cut-off around each MDC in ***Figure 1*** was also arbitrary and may underestimate the population being served by a MDC’s safety net. The retrospective and cross-sectional nature of the study did not account for patient relocation. We may have also inadvertently missed subjects with insurance status changes.

## Conclusions

Geographic disparities are a concern within neurology and neurological disorders. There is a disturbing trend toward increasing subspecialized care concentrated in large tertiary referral centers. Our study methods and conclusions may be applicable beyond MDCs in the state of Florida, potentially translating to other geographically large states in the US, or countries where healthcare is limited by spatial access, like Australia and Canada [18,19]. They also apply to countries without universal healthcare, in which access to healthcare is highly dependent on sociodemographic factors, and access to subspecialized neurological care is limited. Our results highlight the need for increased education and awareness of available movement disorders expertise. The results also highlight the lack of movement disorders specialty care being delivered to underserved populations.

The increased utilization of telemedicine may provide an opportunity to improve access to movement disorders care in the underserved population [20,21,22,23]. Telemedicine does; however, possess its own unique barriers for persons with low socioeconomic status. These include affordability of devices, access to internet and connecting at reasonable hours [24]. House-calls could also be an option; however, the logistics and cost considerations would make this approach challenging [25,26]. Telemedicine should be explored to bring care into the home but there are significant potential barriers in underserved populations.
